# A Network Pharmacology Approach to Investigate the Active Compounds and Mechanisms of Musk for Ischemic Stroke

**DOI:** 10.1155/2020/4063180

**Published:** 2020-07-03

**Authors:** Changlin Zhang, Yingdi Liao, Lingling Liu, Yifan Sun, Shaoqin Lin, Jiaying Lan, Hui Mao, Haoxuan Chen, Yuanqi Zhao

**Affiliations:** ^1^The Second Clinical School, Guangzhou University of Chinese Medicine, Guangzhou, China; ^2^The Second Affiliated Hospital of Guangzhou University of Chinese Medicine, Guangzhou, China; ^3^State Key Laboratory of Dampness Syndrome of Chinese Medicine, The Second Affiliated Hospital of Guangzhou University of Chinese Medicine, Guangzhou, China; ^4^Department of Neurology, Guangdong Provincial Hospital of Chinese Medicine, Guangzhou, China

## Abstract

**Objectives:**

This study aims to study the material basis and effective mechanism of musk for ischemic stroke (IS) based on the network pharmacology approach.

**Methods:**

We collected the chemical components and target gene of musk from the BATMAN-TCM analytical platform and identified ischemic stroke-related targets from the following databases: DisGeNET, NCBI-Gene, HPO, OMIM, DrugBank, and TTD. The targets of musk and IS were uploaded to the String database to construct the protein-protein interaction (PPI) network, and then, the key targets were analyzed by topological methods. At last, the function biological process and signaling pathways of key targets were carried out by Gene Ontology (GO) and Kyoto Encyclopedia of Genes and Genomes (KEGG) pathway enrichment analysis and cluster analysis by using the Database for Annotation, Visualization, and Integrated Discovery (DAVID) server and Metascape platform.

**Results:**

A total of 29 active compounds involving 1081 predicted targets were identified in musk and there were 1104 IS-related targets. And 88 key targets of musk for IS were obtained including AKT1, MAPK1/3, TP53, TNF, SRC, FOS, CASP3, JUN, NOS3, and IL1B. The GO and KEGG enrichment analysis suggested that these key targets are mainly involved in multiple pathways which participated in TNF signaling pathway, estrogen signaling pathway, prolactin signaling pathway, neurotrophin signaling pathway, T-cell receptor signaling pathway, cAMP signaling pathway, FoxO signaling pathway, and HIF1 signaling pathway.

**Conclusion:**

This study revealed that the effective mechanisms of musk against IS would be associated with the regulation of apoptosis, inflammatory response, and gene transcription.

## 1. Introduction

Stroke, an acute cerebrovascular event caused by brain arterial or venous thrombosis or intracerebral hemorrhage, presents a variety of clinical syndromes such as facial paralysis, skewed mouth, slurred speech, or paralysis [1]. The death would happen without timely diagnosis and treatment or serious disability left over due to a large infarct/hemorrhage area. According to a study of the global burden of disease in 2016, the results showed that stroke has been the second cause of mortality and there are 80.1 million stroke patients worldwide, of which approximately 84.4% are ischemic stroke [2]. However, stroke has been the leading cause of premature death in China [3] and causes a serious social burden which may still increase as the persistent aging population and lack of attention to high-risk factors [4]. Currently, clinical therapy includes intravenous thrombolysis, endovascular therapy, antiplatelet aggregation, and other types of management of high-risk factors. Yet, for all this, the mortality has generally declined, and disability and recurrence rates remain high [2], which causes the occurrence of alternative and complementary therapy [5]. Therefore, it is critical to seek more effective measures to prevent and treat stroke.

Chinese herbal medicine has treated stroke for a long time and could provide a rosy prospect for stroke because of rich drugs and prescription resources [6, 7]. Musk (scientific name: *Moschus*, Chinese pinyin: Shexiang) was first set out in *Shen Nong's Materia Medica* and could treat stroke because of the effect of inducing resuscitation, activating blood, dissipating stagnation, and alleviating pain [8]. *The Guide* recorded that musk could clear orifices from our muscles into the bone marrow.* Compendium of Materia* Medica says that it can open contained orifices, channels, and collaterals [9, 10]. Preclinical studies revealed that musk can improve the behavior score and the volume of cerebral infarction in rats with transient local cerebral ischemia-reperfusion injury [11] and showed that muscone could play a neuroprotective role by downregulation of the expression of glutamate transporter and suppression of its reverse transport [12]. Therefore, the extraction of musk has been recommended and listed in China and pharmacopeia for cardiovascular and cerebrovascular disease [13]. However, the mechanism of musk for ischemic stroke was unclear, lacking systematic experiments. So, it is momentous to explore the effective mechanism of musk for ischemic stroke with multiple components, multiple targets, and multiple pathways.

Hopkins first set out that “network pharmacology has become a new scientific method that combined biology, pharmacology, bioinformatics, and computer technology to systematically study the effective mechanisms of multicomponent and multitarget drugs” [14]. This method of analyzing the drug effect mechanism from the network level is an effective supplement to the previous model of reductionist drug development. Chinese medicine has the characteristics of multiple components, multiple targets, and multiple pathways, which is consistent with the research ideas and methods of network pharmacology [15]. This study aimed to use this novel way to predict the musk target, construct the protein-protein interaction (PPI) network, screen topology, and do the gene function analysis. So, based on this approach, it could reveal the mechanisms of musk in treating ischemic stroke to provide evidence of pharmacological experiments and clinical application.

## 2. Materials and Methods

**Figure 1:**
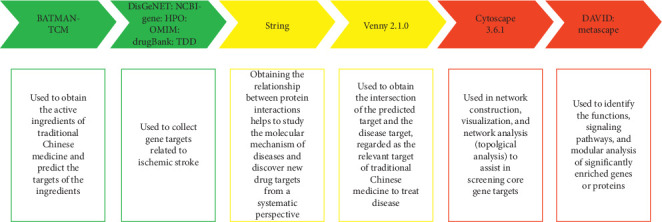
A time table about the use of all databases.

**Figure 2:**
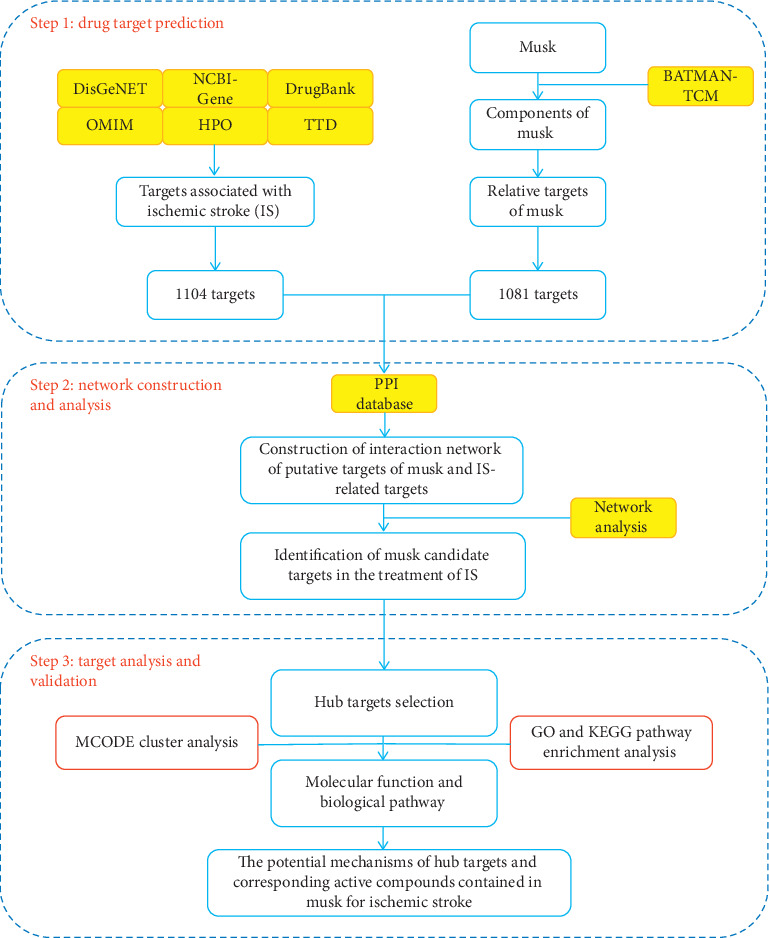
A flow chart of the technical strategy used in this study.

Network pharmacology mainly includes data acquisition and network analysis. The data obtained includes active ingredients of traditional Chinese medicine, a target of component action, and a target of disease-related genes. Based on the data obtained, network analysis constructs and analyzes the interaction between the nodes of the network, the network topology characteristics, and the identification of biological processes and signal pathways affected by the target, thereby clarifying the mechanism of action of traditional Chinese medicine. The following is the role of the database used in the study, as well as the flow chart (see Figures 1 and 2 for details).

### 2.1. Recognizing Compounds of Musk and Predicting Functional Targets

In this study, the BATMAN-TCM analytical platform was used to obtain the composition of musk and to predict the target of the composition. BATMAN-TCM is an online analysis platform for the Molecular Mechanism of Traditional Chinese Medicine. It mainly performs target prediction, target gene function analysis, pathway enrichment analysis, and multitraditional Chinese medicine comparative analysis [16]. It uses a similarity-based method to predict potential targets of herb's ingredients. The principle of this method is to rank all candidate targets according to the prediction score given by the target prediction algorithm for the drug-target interaction prediction, and the targets with higher scores are regarded as potential drug targets [17]. If the score of a predicted target is greater than or equal to the “Score cutoff,” then this target will be regarded as a potential target of the investigated component. So, in the BATMAN-TCM platform “Cluster name,” we input the Pinyin “SHE XIANG” of musk and set the “Score cutoff” value to 20 and adjusted the *P* value which was set at 0.05 to predict the potential target of musk. Then, we used the UniProt (https://www.uniprot.org/) database to search for the predicted target, selected the species as “Homo sapiens,” and excluded nonhuman sources and nonstandard targets. Finally, the relevant targets of musk active ingredients were obtained.

### 2.2. Obtaining Ischemic Stroke-Associated Targets

Ischemic stroke-related targets are collected from the following six disease-related databases: (1) DisGeNET (http://www.disgenet.org) [18], (2) NCBI-Gene (https://www.ncbi.nlm.nih.gov/gene/), (3) Human Phenotype Ontology (HPO) (https://hpo.jax.org/app/) [19], (4) Online Mendelian Inheritance in Man (OMIM) (https://www.omim.org/) [20], (5) DrugBank (https://www.drugbank.ca/) [21], and (6) Therapeutic Target Database (TDD) (http://bidd.nus.edu.sg/group/cjttd/) [22]. In these databases, we use the keywords “ischemic stroke,” “brain ischemia,” “cerebral infusion,” and “cerebral ischemia” as queries to search for known targets associated with ischemic stroke and to remove duplicate genes.

### 2.3. Protein-Protein Interaction (PPI) Network Construction

String (https://String-db.org/) database is a database used to search for protein interactions. It contains a large number of known or predicted protein-protein interactions, including both direct physical interactions and indirect functional relationships [23]. Targets of musk and ischemic stroke were introduced into the String database, and the species was defined as “Homo Sapiens.” The target PPIs of musk and ischemic stroke were constructed. Drawing Venn diagrams is applied online at the intersection of LZTB targets obtained via BATMAN-TCM and Ra targets obtained by DisGeNET. According to the intersection of PPI data, after merging these two networks into candidate networks, the core PPI networks are screened by analyzing the topological characteristics. Venny 2.1.0 (http://bioinfogp.cnb.csic.es/tools/Venny/index.html) was used to analyze the intersection of musk component target and ischemic stroke target PPI. Based on the results of the PPI network intersection, the candidate PPI network was determined, and the target core PPI network of musk-ischemic stroke was further screened through the network topology characteristics.

The following networks were constructed in this study: (1) compound-compound target network of musk, (2) compound target-ischemic stroke network, and (3) a complex network of “compound-target-pathway”. All the networks were generated by employing the network visualization software Cytoscape (version: 3.6.1, http://www.cytoscape.org/). Cytoscape is an open-source software, which conveniently offers numerous bioinformatic analysis tools to further network analysis.

### 2.4. Network Topology Analysis

Network Centrality is a key index to measure the importance of nodes in a network, including degree, betweenness, and closeness centrality [24]. The higher the value of these three indexes is, the more important the node is in the network. This paper uses the “Network Analyzer” plug-in of Cytoscape 3.6.1 to analyze the candidate PPI network and obtains the topological characteristic values such as degree centrality, medium centrality, and closeness centrality. Based on the results of the “Network Analyzer,” the median of degree centrality, medium centrality, and compactness centrality was used as the chi-square value, and the target of satisfying three chi-square values at the same time was selected as the core target for treating ischemic stroke.

### 2.5. Gene Ontology (GO) and KEGG Pathway Enrichment Analysis

Gene Ontology (GO; http://www.geneontology.org/) provides detailed annotation of genes from cell components, molecular functions, and biological processes [25]. Kyoto Encyclopedia of Genes and Genomes (KEGG; https://www.kegg.jp/) provides integration of chemical, genomic, and systemic functional information for understanding the advanced functions and uses of biological systems at the molecular level [26]. The Database for Annotation, Visualization, and Integrated Discovery (DAVID; http://www.david.niaid.nih.gov) integrates GO, KEGG, UniProt, and DrugBank, among other authoritative data sources, to provide systematic bioinformatic annotations for large-scale genes or proteins, including annotation of biological processes and enrichment of pathways [27]. In this study, the core targets of musk for ischemic stroke were uploaded to the DAVID database, which analyzed their gene function and pathway enrichment. According to the *P* value, the core targets were displayed in the form of a chart.

### 2.6. Identifying Core Clusters of PPI Network

In complex biological information networks, some genes or proteins are closely related and have the same or similar functions, so they can be regarded as a cluster and play an important biological role in coordination. The information of each node in the network helps us to perform cluster analysis and build functional modules [28]. Molecular Complex Detection (MCODE) is an automated method for finding molecular complexes in large protein interaction networks. The functional modules of the PPI network in the heart of ischemic stroke treated with musk were selected by using the MCODE analysis function integrated into the Metascape (http://Metascape.org/) [29].

## 3. Results

### 3.1. Compounds of Musk and Target Prediction

According to the BATMAN-TCM database, we selected the chemical composition and target information of musk with the Score cutoff value greater than 20. Among them, 29 chemical constituents of musk were obtained, including macrocyclic ketone compounds such as muscone and normuscone, steroid compounds such as androst-4-ene-3, 17-dione, muscol, and 17-beta-estradiol, and pyridine compounds such as muscopyridine and hydroxymuscopyridine a/b (for more detailed information, see Table 1). In addition, using the principle of chemical structure similarity to predict the target of musk components, 1081 targets were picked up.

### 3.2. Disease Targets

By means of the six databases, namely, DisGeNET, NCBI-Gene, DrugBank, HPO, OMIM, and TTD, we obtained 737, 642, 4, 67, 5, and 16 ischemic stroke-related targets. Summarizing the results of various databases and removing duplicate genes, finally, a total of 1104 ischemic stroke-related targets were picked up.

### 3.3. Construction of PPI Network and Analysis of Network Topological Eigenvalues

**Figure 3:**
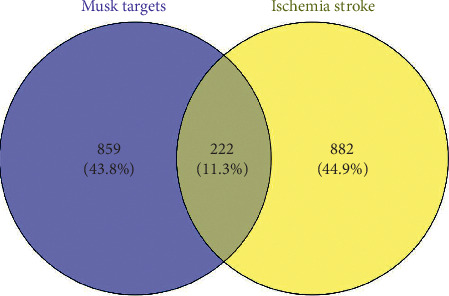
The veen map of targets' intersection of musk and ischemic stroke.

In the current study, we constructed the following networks: compound-compound target network of musk, and compound target-ischemic stroke network. Furthermore, 222 common targets were identified from the above PPI network, as candidate targets (see Figure 3).

**Figure 4:**
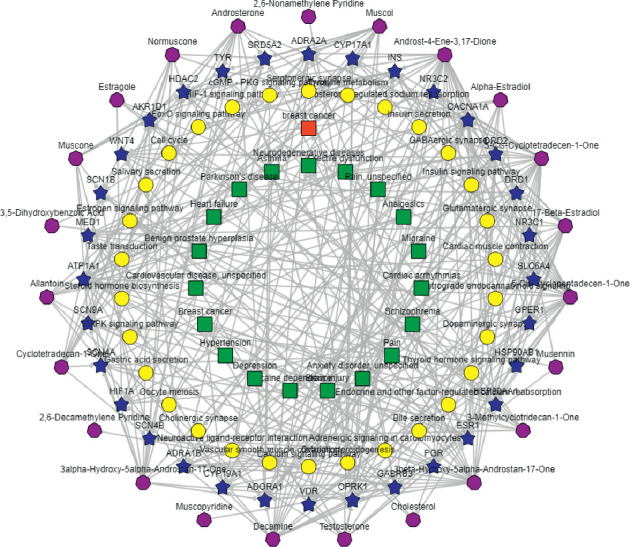
“Ingredient-target-pathway/disease network” of musk (the heptagon is on behalf of the musk compounds, the star node represents prediction target, the yellow circle represents the enrichment pathway, and the red and green squares represent the disease phenotype).

Based on the BATMAN-TCM database, a single herb, musk, was analyzed in terms of gene function, pathway enrichment, and disease phenotype. On the basis of the results, we drew a simplified association network of “ingredients-target-pathway/disease network,” as shown in Figure 4. The results suggested that effective components of musk could act on the cGMP-PKG signaling pathway, HIF-1 signaling pathway, FoxO signaling pathway, MAPK signaling pathway, calcium signaling pathway, vascular smooth muscle contraction, cell cycle, etc. In the aspect of disease phenotypic enrichment, musk had strong pharmacological effects on neurological disease, cardiovascular disease, respiratory disease, mental disorders, and painful diseases.

**Figure 5:**
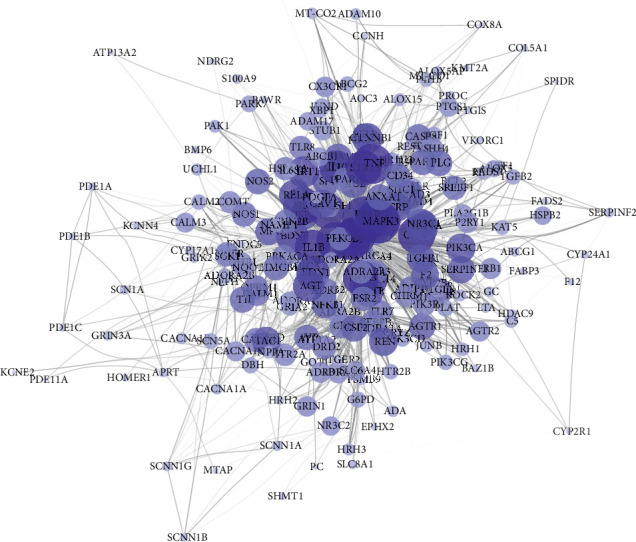
The core target network of musk for ischemic stroke (color and size of nodes reflected degree centrality, while the edge thickness and color's depth reflected the combined score).

After a topological analysis of the PPI network, we chose the target which satisfies the median of degree centrality, medium centrality, and compactness centrality, as core target in treating ischemic stroke. A total of 88 key targets were obtained. Next, we used Cytoscape 3.6.1 software to visualize the component target-disease target interaction network. Network details are shown in Figure 5. Targets with the highest degree of freedom were AKT serine/threonine kinase 1 (AKT1), mitogen-activated protein kinase 1/3 (MAPK1/3), tumor protein p53 (TP53), tumor necrosis factor (TNF), SRC proto-oncogene (SRC), Fos proto-oncogene (FOS), caspase 3 (CASP3), Jun proto-oncogene (JUN), nitric oxide synthase 3 (NOS3), and interleukin 1 beta (IL1B). These results indicated that the mechanism of musk for ischemic stroke was significantly related to those targets.

### 3.4. Enrichment Analysis of Core Targets

In order to understand the function and the underlying significance of core targets, we uploaded 88 core targets into the DAVID database. The result was classified into three parts: biological processes, molecular function, and cellular component. Moreover, those terms were sorted according to the *P* value (see Table 2). In the biological processes, we found that they mainly concentrate on positive regulation of transcription from RNA polymerase II promoter, positive regulation of transcription, DNA-templated, aging, response to drug, positive regulation of gene expression, regulation of blood pressure, response to hypoxia, response to hypoxia, negative regulation of the apoptotic process, and so on. The molecular function was related to transcription factor binding, enzyme binding, protein binding, protein kinase binding, and transcription regulatory region DNA binding. Finally, the cellular component was mainly composed of nucleoplasm, cytosol, protein complex, plasma membrane, extracellular space, caveola, and nucleus.

According to the literature and KEGG enrichment analysis, the pharmacological effects of musk for ischemic stroke were associated with TNF signaling pathway, estrogen signaling pathway, prolactin signaling pathway, neurotrophin signaling pathway, T-cell receptor signaling pathway, cAMP signaling pathway, FoxO signaling pathway, HIF-1 signaling pathway, nonalcoholic fatty liver disease (NAFLD), Rap1 signaling pathway, and so on (the details are depicted in Table 3). This indicated that active components of musk were distributed in different metabolic pathways. Multiple components and multiple targets were the possible mechanisms of musk for ischemic stroke.

### 3.5. Identification of Core PPI Clusters

**Figure 6:**
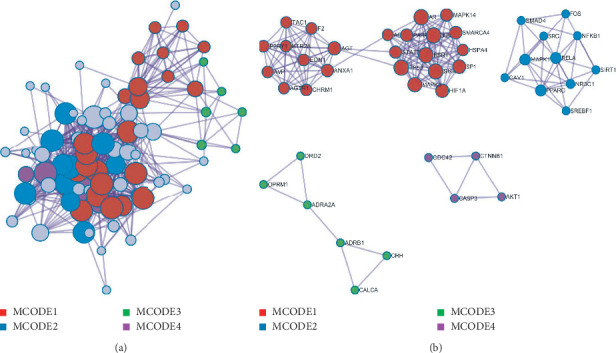
Core network clustering diagram of musk for ischemic stroke.

Based on the MCODE clustering analysis, the core PPI network of musk for ischemic stroke could be divided into 4 modules (Figure 6) as follows. Cluster 1 (MCODE 1) contained 24 genes, score 5.21, and core gene was TP53; cluster 2 (MCODE 2) contained 11 genes, score 2.81, and core gene was RELA; cluster 3 (MCODE 3) contained 6 genes, score 1.17, and core gene was CRH; cluster 4 (MCODE 4) contained 4 genes, score 1.25, and core gene was CTNNB1.

**Figure 7:**
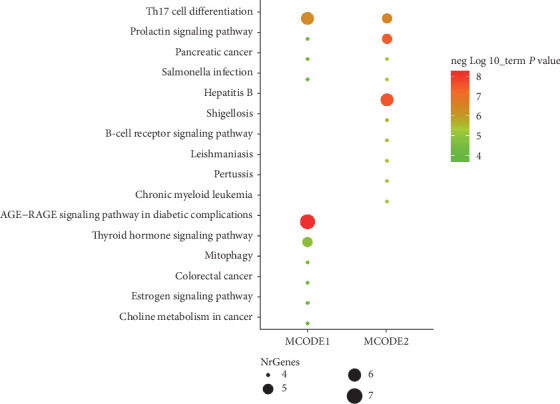
KEGG enrichment analysis of core PPI clusters.

KEGG enrichment analysis of cluster 1 (MCODE 1) and cluster 2 (MCODE 2) was carried out. Top 10 KEGG enrichment pathways were obtained and framed in a bubble plot according to the *P* value (Figure 7). As depicted in the figure, those genes of cluster 1 and cluster 2 were associated with the AGE-RAGE signaling pathway in diabetic complications, Th17 cell differentiation, thyroid hormone signaling pathway, prolactin signaling pathway, and B-cell receptor signaling pathway.

### 3.6. Multidimensional Network Analysis of “Traditional Chinese Medicine-Composition-Target-Pathway”

**Figure 8:**
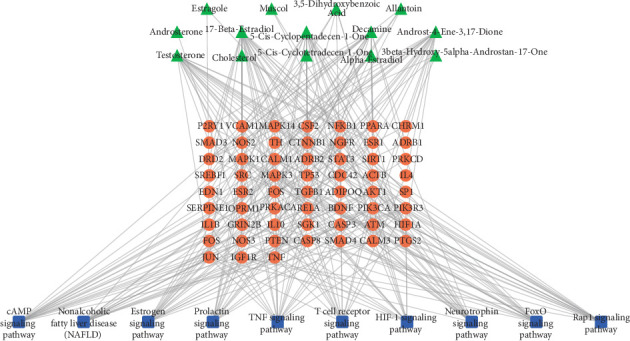
Multidimensional network of “core component-target-pathway.”

Integrating the results of network pharmacological analysis, we constructed a multidimensional network of “traditional Chinese medicine-composition-target-pathway,” as shown in Figure 8. We concluded that the mechanism of musk-mediated ischemic stroke therapy was related to many genes and compounds. It possibly affected AKT1, MAPK1/3, TP53, TNF, SRC, FOS, CASP3, JUN, NOS3, and IL1B. Moreover, musk prevented ischemic stroke via numerous signaling pathways, including TNF signaling pathway, estrogen signaling pathway, prolactin signaling pathway, neurotrophin signaling pathway, T-cell receptor signaling pathway, cAMP signaling pathway, FoxO signaling pathway, and HIF-1 signaling pathway. Therefore, our research had shown that musk can treat ischemic stroke through regulating inflammation-immune response, apoptosis, signal transduction, nervous system, and circulatory system-related signal pathway.

## 4. Discussion

According to TCM theory, ischemic stroke belongs to the category “apoplexy” and causes sudden faint flutter, deviation of the eye and mouth, hemiplegia, etc. Cerebral blood stasis was basic pathogenesis. Therefore, clinical treatments are mainly based on inducing resuscitation, promoting blood circulation by removing blood stasis, and the effect is significant. Chinese medicine “musk” has resuscitation, promoting blood, dissipating mass, and analgesic effect, commonly used in the treatment of ischemic stroke. Currently, basic and clinical research on musk for ischemic stroke mostly focuses on a single component or a single pharmacological effect. However, the pathophysiology of ischemic stroke is complicated. Musk plays a role in the treatment of ischemic stroke through multiple links and multiple ways, and those studies cannot fully reflect that. Studies on system biology have revealed a high degree of correlation between molecular network regulation and diseases. Especially for complex systemic diseases, the simultaneous intervention of multiple targets may achieve better efficacy and less toxic and side effects. Network pharmacology is based on the “disease-gene-target-drug” interaction network, which systematically observes the intervention and impact of drugs on disease networks, thereby revealing the mystery of the synergistic effect of drugs on the human body [30]. Taking ischemic stroke as an example, Casas et al. [31] predicted the protein metabolism network related to NOX4 target from the network pharmacological mechanism and screen core proteins by Gene Ontology analysis. Finally, in vivo and in vitro experiments verified that NOX4 and NOS inhibition is highly synergistic. By inhibiting multiple related targets at the same time, it plays a role in neuroprotection and stabilization of the blood-brain barrier. It can be seen that network pharmacology is of great significance to reveal the mechanism of multitarget drugs to treat diseases. In consequence, this study explores the potential active ingredients and effective mechanisms of musk for ischemic stroke through network pharmacological analysis, with a view to providing theoretical evidence for the development of musk as an adjuvant for ischemic stroke.

In the current study, a total of 29 ingredients including macrocyclic ketone compounds such as muscone, muscol, and normuscone, steroid compounds such as androst-4-ene-3, 17-dione, testosterone, and 17-beta-estradiol, pyridine compounds such as muscopyridine and hydroxymuscopyridine a/b were identified as potential active ingredients of musk. Some active ingredients have proved to be effective in reducing brain damage and improving neurological function after ischemic stroke. For example, muskone can inhibit the expression of Fas and caspase-8 in the cortex, thereby inhibiting apoptosis of neural cells and significantly reducing the volume of cerebral infarction [32]. In addition, after muskone was administered, neural stem cells proliferated actively and transformed into neurons in ischemia-reperfusion rats, which suggested the role of muskone in nerve repair after ischemic stroke [33]. Changes in hormone levels can affect the occurrence of cardiovascular and cerebrovascular diseases [34]. The lower endogenous testosterone concentration in men is associated with a risk of ischemic stroke, which is partially mediated by BMI and hypertension [35]. However, it is unclear whether the treatment of exogenous testosterone has cardiocerebrovascular protection, and further randomized controlled trials are needed to clarify. Moreover, 17-beta-estradiol can protect the brain from ischemic damage after stroke. Its mechanism is closely related to apoptosis, immune regulation, and antioxidative stress [36]. Gibson et al. found that estrogen can reduce the volume of cerebral infarction [37]. The above studies show the effectiveness and diversity of musk active ingredients in the treatment of ischemic stroke.

After analyzing the PPI network of musk for ischemic stroke, we found that the core targets of musk for ischemic stroke were AKT1, MAPK1/3, TP53, TNF, SRC, FOS, CASP3, JUN, NOS3, IL1B, and so on. These targets were involved in transcriptional regulation, gene regulation, apoptosis regulation, and other biological processes. And the results of our research were consistent with the published studies.

AKT is a serine/threonine protein kinase, which plays an antiapoptotic role by phosphorylating downstream target proteins [38]. There are 3 subtypes of AKT, namely, AKT1, AKT2, and AKT3. And AKT1 is widely expressed in tissues in vivo [39]. Evidence showed that AKT expression and phosphorylation increased in cerebral ischemia-reperfusion injury [40]. AKT has neuroprotective effects. Early activation of AKT can reduce infarct volume after cerebral ischemia-reperfusion injury and improve oxygen supply/consumption balance in local brain tissue [41]. According to researches, many Chinese medicine ingredients can regulate the expression of apoptosis-related proteins through AKT/autophagy, PI3K/AKT, and other pathways. In an ischemic and hypoxic environment, muscone was found to improve ischemia-reperfusion injury by activating the PI3K/AKT pathway and promoting endothelial eNOS phosphorylation [42]. Consequently, those ingredients improve the survival rate of nerve cells and reduce the degree of brain damage [43–45]. In addition, well-functioning collateral circulation improves the clinical prognosis of ischemic stroke, so, angiogenesis may be a potential direction for ischemic stroke. AKT also plays an important role in angiogenesis. It not only regulates the expression of VEGF directly but also regulates the expression of various angiogenic factors indirectly, as an upstream signal of mTOR [46, 47].

At present, MAPK signaling has been extensively studied, and it plays a pivotal role in regulating apoptosis and inflammatory cytokine expression after ischemic stroke [48]. In the early stage of ischemia, the expression of p38 MAPK in neurons and glial cells begins to increase [49]. Then, the activated p38 MAPK promotes the release of inflammatory cytokines and induces the expression of adhesion molecules on vascular endothelial cells. Thus, the blood-brain barrier is damaged, and brain damage is aggravated. On the other hand, inflammatory cytokines, in turn, can promote p38 MAPK activation. In the end, it leads to a vicious cycle and exacerbates the cascade of inflammation [50]. MAPK1 (ERK2), MAPK3 (ERK1), and p38 MAPK have high homology. In ischemic stroke, they participate in the regulation of proinflammatory cytokines such as IL-1*β* and TNF-*α* by activating the MAPK cascade [51]. Therefore, by inhibiting the signal transduction of MAPK, it has potential therapeutic prospects for reducing the inflammatory response and blood-brain barrier destruction after ischemic stroke.

Furthermore, TP53 is a tumor suppressor gene, which is associated with the regulation of cell cycle and apoptosis. When an ischemic stroke occurs, the brain is in ischemia and hypoxic environment, which makes the cells in a stress state, and DNA homeostasis is destroyed [52]. Then, TP53-mediated apoptosis was launched. Gomez-Sanchez reports that TP53 Arg/Arg genotype controls the vulnerability of neuronal apoptosis and is a genetic marker for predicting adverse functional outcomes after stroke [53]. TNF is one of the important inflammatory cytokines, with an effect of microglia activation, promoting adhesion and chemokine expression [54]. It is one of the key causes of nerve cell damage after ischemic stroke. Moreover, SRC is a protein with tyrosine-protein kinase activity. It can regulate the biological functions of bradykinin, coupling factor 6, and vascular endothelial growth factor through the redox effect. Thus, SRC plays a crucial part in cardiovascular and cerebrovascular diseases such as hypertension and stroke [55]. As members of the transcription activation factor AP-1 family, FOS (including c-Fos, FosB, Fra1, and Fra2) and JUN (including c-Jun, JunB, and JunD) can form dimers in various combinations through leucine zipper regions [56]. They have biological functions such as cell proliferation, differentiation, and apoptosis. It has been reported that JNK's antiprotease peptides can exert brain-protective functions by blocking c-Fos transcription and c-Jun activation [57].

Based on the KEGG enrichment analysis, musk was thought to influence some important pathways, which were highlighted to play important roles in anti-ischemic stroke. Those signaling pathways included the TNF signaling pathway, estrogen signaling pathway, prolactin signaling pathway, neurotrophin signaling pathway, T-cell receptor signaling pathway, cAMP signaling pathway, FoxO signaling pathway, and HIF-1 signaling pathway.

The pathophysiology of ischemic stroke is very complicated. And inflammation and immune response are important pathophysiological changes in the progression of ischemic stroke, which involves both innate and adaptive immune systems. After a stroke, damaged nerve cells will induce glial cell activation, peripheral immune cell infiltration, and release of inflammatory mediators. Then, the damage of the blood-brain barrier and the occurrence of cerebral edema is aggravated, causing secondary brain damage [58]. Lee et al. found in animal studies that musk of muskrat has a neuroprotective effect. It can reduce the volume of transient focal cerebral ischemic infarction and improve spontaneous and vestibular sensorimotor dysfunction caused by ischemia. The mechanism is related to inhibiting the expression of COX-2 and exerting an anti-inflammatory response [59]. TNF is an inflammatory factor produced by activating monocytes or macrophages. Existing studies reported that the expression of TNF-*α* is significantly increased in patients with acute ischemic stroke. This suggests that TNF-*α* plays a crucial part in the pathogenesis of ischemic stroke [60]. TNF-*α* affects blood-brain barrier permeability. It can activate glial cells and mediates the expression of adhesion molecules on vascular endothelial cells, so as to promote infiltration of neutrophils [61]. Consequently, selective inhibition of TNF-*α* has potential application prospects in reducing blood-brain barrier disruption and improving neurological prognosis [54]. Clinical trial results show that musk can reduce the level of TNF-*α* in patients with ischemic stroke and improve the effectiveness of treatment [62]. Moreover, in terms of adaptive immunity, T-cell receptors are specific receptors on the surface of T cells that provide a connection between T cells and antigen-presenting cells and play an important part in the immune response function of T cells. Severe brain injury, such as ischemic stroke, will rapidly activate the peripheral immune system and promote T or B lymphocytes, monocytes, and neutrophils to the destroyed area [63]. Although the role of adaptive immunity in neuroinflammation has not been clearly defined, studies have demonstrated that mice lacking T and B cells have a smaller infarct size than normal mice [64]. And, a researcher has found that selective inhibition of T-cell receptors can reduce the infarct volume and promote long-term functional recovery [65].

Estrogen mediates genetic effects by binding to the receptors on the nucleus or cell membrane [66]. In recent years, the protective effect of estrogen on ischemic stroke has received widespread attention [67]. Neuroprotective effects of estrogen are mainly exerted by antagonizing excitatory amino acids, antioxidant stress, dilating blood vessels, and increasing cerebral blood flow [68]. The active ingredient in musk, 17-*β*-estradiol, is one of the main ingredients in estrogen. Evidence showed that 17-*β*-estradiol can reduce the infarct volume and reperfusion injury after stroke and accelerates the recovery of motor and sensory functions [69]. Moreover, prolactin is a cofactor for platelet activation [70]. Hyperprolactinemia may be part of the risk factors for stroke, which mediates thrombosis by enhancing platelet reactivity [71]. Neurotrophins family, including nerve growth factor, brain-derived neurotrophic factor, and neurotrophin-3, play an important role in the repair of neural tissue damage. Becker holds that when cerebral ischemia occurs, brain-derived neurotrophic factor binds to the neurotrophin receptor p75 and then activates the c-JunN-terminal kinase pathway to mediate apoptosis [72]. In animal studies, neurotrophin-3 may promote sensory and motor function recovery after stroke [73]. This indicates that neurotrophin-3 has potential applications in the treatment of stroke. cAMP is the second messenger in the cell, which activates cAMP-dependent protein kinase A (PKA). cAMP/PKA signaling pathway is related to nerve regeneration. Yuan et al. found that musk acupuncture treatment can significantly promote the synthesis and release of cAMP, PKA, and pCREB in the brain tissue of ischemic stroke rats and improve the rehabilitation of motor function [74]. Hypoxia-inducible factor 1 (HIF-1) is a major regulator of cells adapting to the hypoxic environment. It can induce angiogenesis, which participates in many pathophysiological processes. In ischemic stroke, HIF-1-mediated angiogenesis is more mature and stable, and it is a promising measure for the treatment of ischemic stroke [75]. Therefore, the role of musk on the HIF-1 signal in ischemic stroke was still controversial, and experimental studies should be designed to evaluate the role of musk for ischemic stroke.

The ischemic stroke involves multiple pathological processes. Traditional Chinese medicine, musk, exerts its pharmacological effect on ischemic stroke through a “multicomponent, multitarget, multipath” effect mechanism. However, our research may have a few limitations. Further biological research is needed in order to verify the results of the above studies.

## 5. Conclusions

In summary, based on the network pharmacology approach, we explored the material basis and effective mechanism of musk for ischemic stroke. Potential functional ingredients such as muscone, testosterone, 17-*β*-estradiol, and musk pyridine have been discovered in this study. These components may have a neuroprotective role after ischemic stroke by regulating various pathways such as apoptosis, inflammatory response, and gene transcription. Our study explained the improvement effect of musk for ischemic stroke theoretically and helped the development and application of musk for ischemic stroke.

## Figures and Tables

**Table 1:** Active chemical constituents of musk.

No	Compound name	Molecular formula	Molecular weight (g/mol)	CID
1	17-Beta-estradiol	C_18_H_24_O_2_	272.4	5757
2	2, 6-Decamethylene pyridine	C_15_H_23_N	217.4	5316410
3	3, 5-Dihydroxybenzoic acid	C_7_H_6_O_4_	154.1	7424
4	3-Alpha-hydroxy-5alpha-androstan-17-one	C_19_H_30_O_2_	290.4	5879
5	3-Beta-hydroxy-5alpha-androstan-17-one	C_19_H_30_O_2_	290.4	441302
6	3-Methylcyclotridecan-1-one	C_14_H_26_O	210.4	3017257
7	5-cis-Cyclopentadecen-1-one	C_15_H_26_O	222.4	5316264
8	5-cis-Cyclotetradecen-1-one	C_14_H_24_O	208.4	5316269
9	Allantoin	C_4_H_6_N_4_O_3_	158.1	204
10	Alpha-estradiol	C_18_H_24_O_2_	272.4	6432565
11	Androst-4-ene-3, 17-dione	C_19_H_26_O_2_	286.4	6128
12	Androsterone	C_19_H_30_O_2_	290.4	5879
13	Cholesterol	C_27_H_46_O	386.7	5997
14	Cholesteryl ferulate	C_37_H_54_O_4_	562.8	22375086
15	Cyclotetradecan-1-one	C_14_H_26_O	210.4	77153
16	Cyclovirobuxine	C_26_H_46_N_2_O	402.7	260439
17	Decamine	C_30_H_40_Cl_2_N_4_	527.6	10649
18	Estragole	C_10_H_12_O	148.2	8815
19	Hydroxymuscopyridine A	C_16_H_25_NO	247.4	184938
20	Hydroxymuscopyridine B	C_16_H_25_NO	247.4	184939
21	Morin	C_4_H_6_N_4_O_3_	302.2	5281670
22	Musclide A1	C_8_H_18_O_5_S	226.3	5319969
23	Muscol	C_12_H_17_NO	191.3	4284
24	Muscone	C_16_H_30_O	238.4	10947
25	Muscopyridine	C_16_H_25_N	231.4	193306
26	Musennin	C_51_H_82_O_21_	1031.2	441933
27	N-Nonane	C_9_H_20_	128.3	8141
28	Normuscone	C_15_H_28_O	224.4	10409
29	Testosterone	C_19_H_28_O_2_	288.4	6013

**Table 2:** GO analysis was implemented on core targets.

Category	Term	Count	*P* value
GOTERM BP DIRECT	GO:0045944-positive regulation of transcription from RNA polymerase II promoter	36	4.65*E* − 21
GOTERM BP DIRECT	GO:0045893-positive regulation of transcription, DNA-templated	28	2.78*E* − 20
GOTERM BP DIRECT	GO:0007568-aging	16	5.75*E* − 15
GOTERM BP DIRECT	GO:0042493-response to drug	19	1.64*E* − 14
GOTERM BP DIRECT	GO:1902895-positive regulation of pri-miRNA transcription from RNA polymerase II promoter	9	4.48*E* − 14
GOTERM BP DIRECT	GO:0010628-positive regulation of gene expression	17	3.44*E* − 13
GOTERM BP DIRECT	GO:0008217-regulation of blood pressure	11	1.16*E* − 12
GOTERM BP DIRECT	GO:0001666-response to hypoxia	14	4.59*E* − 12
GOTERM BP DIRECT	GO:0051091-positive regulation of sequence-specific DNA binding transcription factor activity	12	6.31*E* − 12
GOTERM BP DIRECT	GO:0043066-negative regulation of the apoptotic process	19	1.53*E* − 11
GOTERM MF DIRECT	GO:0008134-transcription factor binding	19	4.57*E* − 15
GOTERM MF DIRECT	GO:0019899-enzyme binding	19	7.25*E* − 14
GOTERM MF DIRECT	GO:0042802-identical protein binding	24	2.39*E* − 12
GOTERM MF DIRECT	GO:0005515-protein binding	77	2.53*E* − 12
GOTERM MF DIRECT	GO:0019901-protein kinase binding	16	7.65*E* − 10
GOTERM MF DIRECT	GO:0044212-transcription regulatory region DNA binding	13	9.23*E* − 10
GOTERM MF DIRECT	GO:0046982-protein heterodimerization activity	16	1.36*E* − 08
GOTERM MF DIRECT	GO:0003707-steroid hormone receptor activity	8	1.43*E* − 08
GOTERM MF DIRECT	GO:0005102-receptor binding	14	2.98*E* − 08
GOTERM MF DIRECT	GO:0000978-RNA polymerase II core promoter proximal region sequence-specific DNA binding	14	3.18*E* − 08
GOTERM CC DIRECT	GO:0005654-nucleoplasm	39	2.19*E* − 10
GOTERM CC DIRECT	GO:0005829-cytosol	41	2.37*E* − 09
GOTERM CC DIRECT	GO:0043234-protein complex	15	8.82*E* − 09
GOTERM CC DIRECT	GO:0005886-plasma membrane	45	1.05*E* − 08
GOTERM CC DIRECT	GO:0005615-extracellular space	25	1.06*E* − 08
GOTERM CC DIRECT	GO:0005576-extracellular region	27	1.66*E* − 08
GOTERM CC DIRECT	GO:0005901-caveola	8	2.46*E* − 08
GOTERM CC DIRECT	GO:0000790-nuclear chromatin	11	2.77*E* − 08
GOTERM CC DIRECT	GO:0043005-neuron projection	11	1.91*E* − 07
GOTERM CC DIRECT	GO:0005634-nucleus	46	1.36*E* − 05

**Table 3:** KEGG enrichment analysis was performed on core targets.

Category	Term	Count	*P* value
KEGG PATHWAY	hsa04668: TNF signaling pathway	18	3.55*E* − 15
KEGG PATHWAY	hsa04915: estrogen signaling pathway	17	1.87*E* − 14
KEGG PATHWAY	hsa04917: prolactin signaling pathway	14	1.23*E* − 12
KEGG PATHWAY	hsa04722: neurotrophin signaling pathway	16	6.49*E* − 12
KEGG PATHWAY	hsa04660: T-cell receptor signaling pathway	15	7.24*E* − 12
KEGG PATHWAY	hsa04024: cAMP signaling pathway	19	9.88*E* − 12
KEGG PATHWAY	hsa04068: FoxO signaling pathway	16	3.29*E* − 11
KEGG PATHWAY	hsa04066: HIF-1 signaling pathway	14	6.72*E* − 11
KEGG PATHWAY	hsa04932: nonalcoholic fatty liver disease (NAFLD)	15	2.01*E* − 09
KEGG PATHWAY	hsa04015: Rap1 signaling pathway	17	2.26*E* − 09

## Data Availability

The data used to support the findings of this study are available from the corresponding author upon request.

## Supplementary Materials

Supplementary Materials The detailed information of chemical composition and predicted target of musk (S1), ischemic stroke target (S2), string data (S3), compound target analysis data (S4), Gene Ontology enrichment analysis results (S5, S6, and S7), and KEGG enrichment analysis results (S8 and S9) used to support the findings of this study is included within the supplementary information files.
